# The patterns of Non-communicable disease Multimorbidity in Iran: A Multilevel Analysis

**DOI:** 10.1038/s41598-020-59668-y

**Published:** 2020-02-20

**Authors:** Zahra Khorrami, Maysam Rezapour, Koorosh Etemad, Shahin Yarahmadi, Soheila Khodakarim, Alireza Mahdavi Hezaveh, Mohammadesmail Kameli, Narges Khanjani

**Affiliations:** 1 Neurology Research Center, Kerman University of Medical Sciences, Kerman, Iran; 2 Department of Epidemiology, School of Public Health and Safety, Shahid Beheshti University of Medical Sciences, Tehran, Iran; 3 School of Nursing and Midwifery Amol, Mazandaran University of Medical Sciences, Sari, Iran; 4 Endocrine and Metabolic Diseases Office, Center for Noncommunicable Disease Control, Ministry of Health and Medical Education, Tehran, Iran; 5 School of Allied Medical Sciences, School of Public Health and Safety, Shahid Beheshti University of Medical Sciences, Tehran, Iran; 6 Center for Noncommunicable Disease Control, Ministry of Health and Medical Education, Tehran, Iran; 7 Department of Community Medicine, School of Medicine, Iran University of Medical Sciences, Tehran, Iran; 8 Environmental Health Engineering Research Center, Kerman University of Medical Sciences, Kerman, Iran

**Keywords:** Metabolic disorders, Disease prevention, Epidemiology

## Abstract

The prevalence of non-communicable diseases is increasing worldwide. Multimorbidity and long-term medical conditions is common among these patients. This study aimed to investigate the patterns of non-communicable disease multimorbidity and their risk factors at the individual and aggregated level. Data was inquired from the nationwide survey performed in 2011, according to the WHO stepwise approach on NCD risk factors. A latent class analysis on multimorbidity components (11 chronic diseases) was performed and the association of some individual and aggregated risk factors (urbanization) with the latent subclasses was accessed using multilevel multinomial logistic regression. Latent class analysis revealed four distinct subclasses of multimorbidity among the Iranian population (10069 participants). Musculoskeletal diseases and asthma classes were seen in both genders. In males, the odds of membership in the diabetes class was 41% less by increasing physical activity; but with increased BMI, the odds of membership in the diabetes class was 1.90 times higher. Tobacco smoking increased the odds of membership in the musculoskeletal diseases class, 1.37 and 2.30 times for males and females, respectively. Increased BMI and low education increased the chances of females’ membership in all subclasses of multimorbidity. At the province level, with increase in urbanization, the odds of membership in the diabetes class was 1.28 times higher among males (P = 0.027). Increased age, higher BMI, tobacco smoking and low education are the most important risk factors associated with NCD multimorbidity among Iranians. Interventions and policies should be implemented to control these risk factors.

## Introduction

The World Health Organization (WHO) global status report on non-communicable diseases (NCDs) in 2014 reported that NCDs are globally the leading cause of death^1^. In 2016, NCDs killed 287000 people in Iran and the number of NCD related deaths and disability-adjusted life years (DALYs) have increased during the past decades. In just 2016, 6.5 million years of life loss (YLLs), and 8.2 million years of disability (YLDs) were attributed to NCDs in Iran^2^. According to 2017 reports, in the past 20 years, NCD mortality has risen by 14.5%, in Iran; and an adult Iranian’s probability of dying prematurely (between 30 and 70 years) from one of the four main NCDs was 17%^3^.

A systematic review in WHO Eastern Mediterranean countries in 2013 showed that the high mortality of NCDs is partially related to their multimorbidity^4^. More than half of the adults with NCDs have multimorbidity or multiple concurrent morbid conditions, and not one single chronic disease^5^.

The prevalence of multimorbidity is increasing worldwide^6^. NCD multimorbidity affects more young people in low- and middle-income countries. The mean prevalence of multimorbidity was 7.8% in 28 developing countries in 2015^7^. NCD multimorbidity may occur 10–15 years earlier in individuals living in developing countries^8^ and as mean population age increases, the growing prevalence of NCD multimorbidity has led to decreased quality of life among these patients^9–11^. Multimorbidity is already, and will be in the future, a great challenge for both developed and developing countries^12^.

On the basis of WHO estimates, by 2050, Iran, as a developing country will have a high proportion of old citizens^13^. Also Iran’s urban population and people’s life expectancies have risen in the recent years. However, people’s exposure to tobacco, unhealthy diets, and physical inactivity have increased as well^14^.

NCDs are driven by forces that include rapid unplanned urbanization, globalization of unhealthy lifestyles and population ageing. Urbanization is one of the main socioenvironmental factors which causes unhealthy lifestyle, and diet and low physical activity^15^. Hypertension, diabetes, musculoskeletal disorders, obesity, and their risk factors, increase with development, urbanization, lifestyle changes^16,17^ and poverty^18^.

Knowledge about multimorbidity patterns has important implications for prevention, diagnosis, and treatment, in societies, can provide essential information for developing guidelines for patients with multiple chronic conditions^8^ and can help governmental authorities allocate resources for health care appropriately^19^. Epidemiological data on the multimorbidity of NCDs and their risk factors, and consequences are important for designing and developing strategies to reduce the burden of NCDs^20^. However, there is no consensus on which specific conditions should be considered; or on the method used for measuring multimorbidity^21^.

Previous studies have evaluated NCD multimorbidity by using definite cut points such as the Charlson Comorbidity Index or variable-centered approaches (PCA or factor analysis), that categorize people into discrete sub-groups^22–24^.

Considering the possibility that the relation between multimorbidity components may differ in different subclasses significantly, latent class analysis (LCA) has been used to classify similar individuals into groups or latent clusters. In the LCA method, a person-oriented grouping approach is used, which can simultaneously consider the effects of many contexts (race, genetics, society, environment, etc.) for classifying people in latent clusters that are homogeneous groups of individuals in regard to multimorbidity^25^. This method has also been used in previous studies^26^.

In the current study, we aimed to investigate the patterns of multimorbidity among Iranian adults in total and in gender groups. And also determine if the emergent latent classes of multimorbidity differ based on demographic or NCD risk factors.

## Methods

### Study population and survey design

A survey about NCDs was conducted by the Ministry of Health and Medical Education, with a sample size of 10069 people aged 20–70 years, from 31 provinces (7033 from urban and 3036 from rural areas), in 2011. Although the sample size of the STEPS 2011 study in Iran was 12,000 people aged 6 to 70 years. In our study, the age group was limited to 20 to 70 year old people and the sample size was reduced to 10069.

Participants were selected by multistage random cluster sampling. Details about the sampling method has been mentioned elsewhere^27^. For all participants, an NCD risk factor questionnaire adapted from the WHO STEPS program was used to collect information on demographics, NCDs and their risk factors by trained personnel. Details of the methods has been published, previously^28^.

### Measurement of Non-communicable diseases and their risk factors

The data used in this study included information on demographics (age, gender, education, and occupation) and risk factors. Tobacco use was defined as daily cigarette and/or water-pipe use. Fruit and vegetable consumption was defined as number of servings of fruit and vegetables taken per day (less than 3 serving, 3–5 serving, more than 5 serving). Physical activity was defined according to the Global Physical Activity Questionnaire (GPAQ), as minutes spent for physical activity per week, during 3 settings, which were occupation, commuting, and recreational time. Information about these variables is available on the WHO website^29^. Blood pressure measurement and anthropometric measurements were performed by trained medical personnel^15^, and WHO defined cut-off points were used for categorizing these variables^28^.

The non-communicable diseases evaluated in this study were self-reported and included Diabetes, Coronary Artery Disease (e.g., Stroke and heart attack), Osteoporosis, Back Pain, Neck Pain, Knee Pain, Breast Cancer, Colorectal Cancer, Cerebrovascular disease (e.g., Stroke and Cerebral hemorrhage), and Asthma.

### Assessment of community-level factors (urbanization)

We used the “urbanization” index to investigate the effect of the community-level variables (province) in this study. We considered the following community-level factors in our analysis: average household size, population density, urbanization rate, average floor area of the dwelling unit per family member, economic participation rate, unemployment rate, employment in agriculture and industry, internet penetration, telephone and mobile penetration rate, percentage of villages with telephone communication, gas and electricity energy use per 1000 population, percentage of cities and rural areas with gas facilities, proportion of physicians per 1000 population, proportion of nurses per 1000 population, proportion of specialist physicians per 1000 population, and the human development index. These variables were combined to reduce the number of variables and create an index for urbanization. More details have been mentioned in another paper^15^.

### Ethical considerations

This study used data from the 2011 National Survey of NCD Risk Factor (STEPS) conducted by the Ministry of Health and Medical Education of the Islamic Republic of Iran. All respondents were interviewed after signing the consent form. Participant’s identity was kept confidential. This project was approved by the Ethics in Research Committee of Shahid Beheshti University of Medical Sciences.

### Statistical analysis

The analytic strategy in the current study was in two steps. First, we used a latent class analysis to identify homogeneous subgroups based on multimorbidity with NCDs (mentioned above), which have a common pattern of co-morbidity within a heterogeneous population. Second, we examined the relations between some risk factors and class memberships using multilevel multinomial logistic regression. The goal of this second step of the analysis was to determine which individual- or province level characteristic was associated with multimorbidity latent class membership among the nationwide population, while controlling for the nested nature of the data (population within different provinces).

LCA was fit as an exploratory and iterative process with an increasing number of classes. The final number of classes was determined by considering model fitness indices, empirical evidence and interpretability.

Model fitness was assessed using the Akaike Information Criterion (AIC), Bayesian Information Criterion (BIC), Sample Size Adjusted Bayesian Information Criterion (SSABIC), the Lo–Mendell–Rubin likelihood ratio (LMR LR) test, Vuong-Lo-Mendell-Rubin likelihood ratio test (VLMR**)**, and bootstrap likelihood ratio test (BLRT). Smaller values of AIC, BIC, and SSA BIC indicated a better model fit. The LMR is a likelihood ratio test and compares the estimated model to a model with one less class (k-1). If the LMR is not significant, model fitness will not improve by including an additional class into the model^30^. The VLMR and BLRT P-values are interpreted in the same way as the LMR LR test. Entropy was used to assess the quality of member classification; a value closer to 1.0 indicates better classification^31^.

Two-level multinomial logistic regression analysis with random intercept was conducted to explore the association of both individual and province- level predictors with non-communicable disease multimorbidity, in males and females, separately. The “low-risk of disease” category was used as the reference category. Each class was compared with the low-risk class in the same gender.

Initially an empty model with no predictors was built, and then individual-level and province-level variables were added. The variance components (random effects) of outcomes in both models for males and females were estimated as the province-level variance.

Data preparation was done in Stata14; latent class analysis and regression models were fit in Mplus 7.4.

In this study the full-information maximum likelihood (FIML) estimation was used, which is recommended for handling missing data under missing at random (MAR) or missing completely at random (MCAR) situations. This method uses the robust maximum likelihood estimator (MLR) which uses model-based methods^32^.

Initially, an empty model with no predictors was fitted to examine the variation of the NCD multimorbidity classes across provinces, for males and females; separately. The variance component and the standard error of provinces in the empty model for males was 0.083 and 0.035 respectively; and the ICC was 2.4%. These values were 0.021, and 0.035 and the ICC was 0.6% in females. These low ICCs in both male and females show that the variation of the NCD multimorbidity classes at province-level is low.

Although, authors suggest that ICCs smaller than 0.05 may benefit less from multi-level analysis, Muthén states that regardless of the ICC, design effects greater than 2 need to take into account multilevel-analysis^33^. In the current study, the design effect was 4.36 for females and 14.44 for males according to this equation:

1 + (average cluster size - 1)*intraclass correlation

Thus, a two-level multinomial logistic regression analysis (random intercept) was conducted to explore the concurrent association of individual and province- level predictors with NCDs, for males and females, separately.

In the first part of the study which aimed at extracting multimorbidity patterns in the Iranian population, there was no missing data. In the second part, which examined the association between multi-morbidity patterns; 20% of the physical activity variable and 28% of the fruit and vegetable consumption variable were missing.

## Results

### Participant characteristics

The descriptive statistics of all variables are shown in Table 1.

**Table 1:** Descriptive statistics of the variables used in this study.

Characteristic	Total (n = 10069) N (%)	Males (n = 4136) N (%)	Females (n = 5933) N (%)	p-value
**Demographic**				
Mean Age(year)(SD)	43 (15.34)	43 (15.49)	43 (15.23)	0.052
Education status				
Illiterate	2583 (25.7)	657 (15.9)	1925 (32.4)	
Elementary	2194 (21.8)	924 (22.3)	1270 (21.4)	
High School	1516 (15.1)	753 (18.2)	763 (12.9)	P < 0.001
Diploma	2294 (22.8)	1062 (25.7)	1232 (20.8)	
Academic	1473 (14.6)	732 (17.7)	741 (12.5)	
Job status				
Government officer	620 (6.2)	409 (9.9)	211 (3.6)	
Private sector employee	654 (6.5)	520 (12.6)	134 (2.3)	
Self-employed	1919 (19.1)	1749 (42.3)	170 (2.9)	P < 0.001
Student or Soldier	658 (6.5)	334 (8.1)	324 (5.5)	
Retiree or unemployed, Housewife, unpaid work	6218 (61.8)	1123 (27.2)	5094 (85.9)	
**Risk factors**				
Hypertension	1706 (16.9)	697 (16.9)	1009 (17.1)	0.916
Physical Activity				
Low	4246 (42.2)	1608 (38.9)	2638 (44.5)	
Moderate	2851 (28.3)	1374 (33.2)	1476 (24.9)	
High	529 (5.3)	400 (9.7)	129 (2.2)	P < 0.001
BMI				
Normal (≤24.9)	4219 (41.9)	1967 (47.6)	2252 (38.0)	
Overweight (25–29.9)	3505 (34.8)	1515 (36.6)	1989 (33.5)	P < 0.001
Obese (≥30)	2288 (22.7)	617 (14.9)	1671 (28.2)	
Daily tobacco use	1328 (13.2)	1093 (26.4)	235 (4.0)	P < 0.001
Fruit & vegetable intake				
<3 servings per day	8448 (83.9)	3434 (83.0)	5013 (84.5)	
3–5 servings per day	200 (2.0)	84 (2.0)	116 (2.0)	0.658
≥5 servings per day	29 (0.3)	14 (0.3)	15 (0.3)	
**Diseases**				
Diabetes	1006 (10.0)	338 (8.2)	668 (11.3)	P < 0.001
Coronary Artery Disease	730 (7.2)	275 (6.7)	455 (7.7)	0.161
Osteoporosis	977 (9.7)	112 (2.7)	865 (14.6)	P < 0.001
Back Pain	2246 (22.3)	599 (14.5)	1646 (27.7)	P < 0.001
Neck Pain	1244 (12.4)	288 (7.0)	956 (16.1)	P < 0.001
Knee Pain	2450 (24.3)	610 (14.8)	1839 (31.0)	P < 0.001
Breast Cancer	80 (0.8)	9 (0.2)	71 (1.2)	P < 0.001
Colorectal Cancer	38 (0.4)	14 (0.3)	24 (0.4)	0.682
Cerebrovascular disease	170 (1.7)	66 (1.6)	104 (1.8)	0.731
Asthma	358 (3.6)	143 (3.5)	215 (3.6)	0.741
Wheezing	530 (5.3)	228 (5.5)	302 (5.1)	0.311

Abbreviation: SD, standard deviation.

BMI = Body mass index was calculated as weight (kg)/height (m)^2^.

Hypertension: Systolic blood pressure ≥140 mmHg and/or diastolic blood pressure ≥90 mmHg.

### Multimorbidity patterns

A series of latent class models were ran in which the number of classes ranged from 2 to 6 for males and 2 to 7 for females. Table 2 shows the goodness-of-fit indices for each model. Although the VLMR and LMR suggested 5-class models for males and females, we selected the 4-class model for both males and females, because it had a smaller BIC, SSABIC and a high entropy value.

**Table 2:** Latent Class Analysis Fit Indices for males and females.

N class	Log-likelihood	AIC	BIC	SSABIC	LMR(P-value)	VLMR(P-value)	Entropy	Bootstrap LR(P-value)
**Male**								
2 class	-8087.884	16217.77	16350.48	16283.75	<0.001	<0.001	0.754	<0.001
3 class	−7998.045	16060.09	16262.31	16160.63	<0.001	<0.001	0.832	<0.001
4 class	−7949.804	15985.61	16257.35	16120.71	<0.001	<0.001	0.738	<0.001
5 class	−7922.112	15952.22	16293.48	16121.89	0.0116	0.0111	0.735	<0.001
6 class	−7905.51	15941.02	16351.79	16145.25	0.4104	0.4047	0.725	<0.001
**Female**								
2 class	−16237.2	32520.31	32674.11	32601.02	<0.001	<0.001	0.667	<0.001
3 class	−16082.9	32235.84	32469.8	32358.67	<0.001	<0.001	0.766	<0.001
4 class	−15965.2	32024.37	32338.66	32189.31	<0.001	<0.001	0.65	<0.001
5 class	−15914.2	31946.31	32340.84	32153.36	0.014	0.0134	0.635	<0.001
6 class	−15892.6	31927.2	32401.98	32176.37	0.0232	0.0224	0.643	<0.001
7 class	−15872.9	31911.76	32466.79	32203.04	0.1511	0.1484	0.651	0.01

Abbreviations: AIC, Akaike Information Criterion; BIC, Bayesian Information Criterion; SSABIC, Sample Size Adjusted Bayesian Information Criterion; LMR, Lo–Mendell–Rubin likelihood ratio test; VLMR, Vuong-Lo-Mendell-Rubin likelihood ratio test; Bootstrap LR, bootstrap likelihood ratio test.

The response probabilities for each of the classes in males and females are displayed in Table 3.

**Table 3:** Disease probabilities per class for males and females.

Males	Class 1	Class 2	Class 3	Class 4
Probability of membership	268 (6.5%)	90 (2.2%)	376 (9.2%)	3369 (82.1%)
Diabetes	**0.54**	0.20	0.11	<0.01
Coronary Artery Disease	0.20	0.19	0.16	0.04
Osteoporosis	0.05	<0.01	0.16	0.01
Back Pain	0.11	0.20	**0.88**	0.08
Neck Pain	0.04	0.24	**0.51**	0.02
Knee Pain	0.16	0.29	**0.81**	0.08
Colorectal Cancer	<0.01	0.02	0.01	<0.01
Cerebrovascular disease	0.06	0.09	0.02	<0.01
Asthma	<0.01	**0.55**	0.06	0.01
Wheezing	<0.01	**0.73**	0.09	0.03
**Females**	**Class 1**	**Class 2**	**Class 3**	**Class 4**
Probability of membership	223 (3.8%)	1479 (25.0%)	695 (11.7%)	3529 (59.6%)
**Conditional probability of a Yes response**				
Diabetes Mellitus	0.21	0.14	0.21	0.05
Coronary Artery Disease	0.27	0.10	0.19	0.03
Osteoporosis	0.36	0.25	0.49	<0.01
Back Pain	0.39	0.38	**0.99**	0.08
Neck Pain	0.20	0.17	**0.83**	0.01
Knee Pain	**0.54**	**0.59**	**0.96**	0.01
Breast Cancer	0.03	0.02	0.03	0.01
Colorectal Cancer	0.02	<0.01	<0.01	0.01
Cerebrovascular disease	0.03	0.02	0.04	0.01
Asthma	**0.58**	<0.01	0.05	0.01
Wheezing	**0.56**	<0.01	0.11	0.02

Conditional probabilities >0.5 in bold to facilitate interpretation.

In males the first class (labeled “Diabetes”), constituted of 6.5% of the male sample, and was characterized by a high probability of diabetes (0.54) and a low probability of other diseases. This group also had the highest probability of Coronary Artery Disease (0.20). Class two (2.2% of the male sample, labeled “Asthma and Wheezing”) was characterized by a high probability of Asthma (0.55) and Wheezing (0.73). The third class (labeled “Musculoskeletal diseases”), comprised 9.2% of the male sample and was characterized by a high probability of Back Pain (0.88), Neck Pain (0.51), and Knee Pain (0.81) and a low probability of other disease. This group also had the highest probability of Osteoporosis (0.16). The fourth class (labeled “Low risk of diseases”), comprised 82.1% of the male sample and was characterized by a low probability of all diseases.

For females the first class (labeled “Asthma and Wheezing”), constituted 3.8% of the female sample, and was characterized by a high probability of Asthma (0.58) and Wheezing (0.56). Class two (25.0% of the female sample, labeled “pre-skeletal diseases”) was characterized by a moderate probability of Osteoporosis (0.25), Back Pain (0.38), Neck Pain (0.17), and Knee Pain (0.59). The third class (labeled “Musculoskeletal diseases”), which comprised 11.7% of the female sample was characterized by a high probability of Back Pain (0.99), Neck Pain (0.83), and Knee Pain (0.96) and a low probability of other disease except Osteoporosis. This group also had the highest probability of Osteoporosis (0.49). The fourth class (labeled “Low risk of diseases”), comprised 59.6% of the female sample and was characterized by a low probability of all diseases. The probability of Knee Pain was high in class 1 to 3. Colorectal Cancer and Cerebrovascular disease had a low probability in all male and female classes.

### Multimorbidity and multilevel analysis

The variance component and the standard error of provinces in the empty model for males was 0.083 and 0.035 respectively; and the ICC was 2.4%. These values were 0.021, and 0.035 and the ICC was 0.6% in females. These low ICCs in both male and females show that the variation of the NCD multimorbidity classes at province-level is low. In the current study, the design effect was 4.36 for females and 14.44 for males.

Table 4 shows the association between the predictors and the NCD multimorbidity classes in males. At individual-level, the odds of membership in all classes (class 1 to class 3) compared to the low-risk of disease class were higher as age increased. The hypertensive individuals had a higher odds for membership in class 1 (Diabetes) compared to the low-risk class (OR = 1.59; 95% CI: 1.15–2.21). The odds of membership in the diabetes class compared to the low-risk class was 41% less by increasing physical activity from one level to the other (levels: low, moderate, and high). With one level increase in BMI (levels: normal, over-weight, and obese), the odds of membership in the diabetes class was 1.90 (95% CI: 1.45–2.49) times higher. Comparing the tobacco-using individuals to the non-using individuals, the odds of membership in the musculoskeletal diseases class versus the low-risk class was 1.37 times higher.

**Table 4:** Odds Ratios and 95% Confidence Intervals of the association between Non-communicable Multimorbidity Classes and Predictors in Individual- and province-Level using Two-Level Multinomial Logistic Regression Analysis for males.

	Diabetes	Asthma and Wheezing	Musculoskeletal diseases
***Individual-Level***	OR (95% CI)	OR (95% CI)	OR (95% CI)
Age	1.06 (1.05, 1.07)***	1.04 (1.01, 1.07)**	1.03 (1.02, 1.05)***
Hypertension	1.59 (1.15, 2.21)**	1.28 (0.79, 2.07)	0.94 (0.63, 1.38)
Physical Activity	0.59 (0.45, 0.76)***	0.86 (0.52, 1.42)	1.07 (0.81, 1.41)
BMI	1.90 (1.45, 2.49)***	1.04 (0.67, 1.62)	1.14 (0.97, 1.34)
Daily tobacco use	0.73 (0.42, 1.26)	0.94 (0.49, 1.82)	1.37 (1.04, 1.80)*
Fruit and vegetables intake	1.31 (0.58, 2.98)	0.62 (0.17, 2.31)	0.81 (0.25, 2.56)
**Education level (Ref: Academic education)**			
Illiterate	0.99 (0.55, 1.77)	1.28 (0.53, 3.10)	1.96 (1.16, 3.31)*
Elementary	2.00 (1.31, 3.05)***	0.96 (0.55, 1.69)	1.48 (0.92, 2.40)
High School	1.66 (0.84, 3.26)	1.15 (0.50, 2.64)	1.00 (0.52, 1.92)
Diploma	1.20 (0.71, 2.04)	0.47 (0.15, 1.49)	0.95 (0.57, 1.57)
**Job (Ref: government officer)**			
Private sector employee	0.63 (0.31, 1.29)	1.00 (0.29, 3.46)	0.74 (0.41, 1.36)
Self_employed	0.68 (0.45, 1.04)	0.95 (0.48, 1.9)	0.85 (0.48, 1.51)
Student or Soldier	0.38 (0.10, 1.39)	0.72 (0.13, 3.92)	0.42 (0.18, 0.98)*
Retiree, unemployed, Housewife, unpaid work	0.81 (0.46, 1.42)	0.74 (0.31, 1.78)	0.85 (0.43, 1.68)
***Province -Level***			
Urbanization	1.28 (1.03, 1.59)*	0.89 (0.56, 1.41)	0.98 (0.81, 1.19)
***Variance component of provinces (SE)***	0.085 (0.055)		
***ICC %***	2.5%		

*P-value < 0.05, **P-value < 0.01, ***P < 0.001.

Illiterate individuals compared with those with academic education had a higher odd for membership in the musculoskeletal diseases class (OR = 1.96; 95% CI: 1.16–3.31). Also, individuals with elementary-level education had higher odds for membership in the diabetes class compared with individuals who had academic education (OR = 2.00; 95% CI: 1.31–3.05). Students and soldiers compared with governmental officers had a 58% less odds for membership in the skeletal-diseases class versus the low-risk class. At province-level, with one unit increase in the urbanization score, the odds of membership in the diabetes class were 1.28 times higher.

Table 5 shows the associations between the predictors and the NCD multimorbidity classes in females. At individual-level, the odds of membership in all classes (1 to 3) were higher as age increased. Hypertensive individuals had a lower odds for membership in class 2 (OR = 0.77; 95% CI: 0.61–0.98). With one level increase in BMI (levels: normal, over-weight, and obese), the odds of membership in all classes versus the low-risk class increased. Among tobacco-using individuals, the odds of membership in the Musculoskeletal diseases class was 2.30 (95% CI; 1.35–3.93) times higher. Illiterate individuals had higher odds for membership in all classes compared with those who had academic education. Also, the individuals with elementary education had higher odds for membership in class 1 and 2 compared with those who had academic education. But the individuals with a high school diploma had higher odds for membership only in class 2 compared with those who had academic education. Retirees or un-employed people had a 54% less odds for membership in the musculoskeletal diseases class compared to governmental officers. Private employees had very low odds for membership in the asthma and wheezing class compared to governmental employees. At province-level, urbanization score was not associated with NCD multimorbidity in females.

**Table 5:** Odds Ratios and 95% Confidence Intervals of the Association Between Non-communicable Multimorbidity Classes and Predictors in Individual- and provinces-Level Using Two-Level Multinomial Logistic Regression Analysis for females.

*Individual-Level*	Asthma and Wheezing	Pre-skeletal diseases	Musculoskeletal diseases
	OR (95% CI)	OR (95% CI)	OR (95% CI)
Age	1.04 (1.03, 1.06)***	1.07 (1.06, 1.08)***	1.08 (1.07, 1.10)***
Hypertension	0.84 (0.54, 1.30)	0.77 (0.61, 0.98)*	0.81 (0.57, 1.17)
Physical Activity	1.13 (0.78, 1.66)	1.07 (0.88, 1.32)	1.02 (0.78, 1.33)
BMI	1.58 (1.26, 1.99)***	1.25 (1.12, 1.40)***	1.41 (1.20, 1.65)***
Daily tobacco use	2.10 (0.96, 4.59)	1.20 (0.80, 1.80)	2.30 (1.35, 3.93)**
Fruit and vegetables intake	1.32 (0.71, 2.42)	1.08 (0.80, 1.46)	1.20 (0.69, 2.07)
**Education level (Ref: Academic education)**			
Illiterate	7.8 (3.07, 19.84)***	2.11 (1.51, 2.95)***	1.85 (1.14, 3.01)*
Elementary	5.24 (1.95, 14.05)***	1.82 (1.31, 2.52)***	1.35 (0.79, 2.31)
High School	2.87 (0.98, 8.41)	1.47 (0.93, 2.32)	1.35 (0.72, 2.53)
Diploma	2.45 (0.69, 8.64)	1.57 (1.07, 2.3)*	0.85 (0.4, 1.84)
**Job (Ref: government officer)**			
Private sector employee	0.00 (0.00, 0.01)***	1.28 (0.64, 2.54)	0.41 (0.15, 1.14)
Self-employed	1.87 (0.44, 7.98)	1.19 (0.69, 2.06)	0.34 (0.07, 1.65)
Student or Soldier	2.78 (0.57, 13.55)	0.54 (0.29, 1.03)	0.52 (0.20, 1.35)
Retiree, unemployed, Housewife, unpaid work	1.75 (0.38, 8.12)	0.82 (0.51, 1.33)	0.46 (0.26, 0.82)**
***Province -Level***			
Urbanization	0.94 (0.76, 1.15)	0.90 (0.79, 1.03)	0.79 (0.60, 1.05)
***Variance component of Provinces (SE)***	0.064 (0.024)		
***ICC %***	1.9%		

*P-value < 0.05, **P-value < 0.01, ***P < 0.001.

The variance in the non-communicable multimorbidity classes in both males and females at the province-level did not increase significantly after including individual-level and province-level in the model.

## Discussion

The current study was conducted by LCA and provided evidence of NCD multimorbidity in four classes, for the 20 to 70 year old population of Iran, in both males and females. These classes were diabetes (6.5%), asthma (2.2%), musculoskeletal diseases (9.2%), and low-risk of NCD (82.1%) for males; and asthma (3.8%), pre-skeletal diseases (25.0%), musculoskeletal diseases (11.7%), and low-risk of NCD (59.6%) for females.

Although the Charlson Comorbidity Index has also been used in similar research, but it was not appropriate for this study, because it includes 17 morbidities and this study did not have data for all of them. On the other hand, the LCA approach, takes into account response measurement errors and provides several goodness of fit indexes, and therefore provides more realistic co-morbidity patterns for the same population, at the same time, which is an advantage. Results are more reliable in LCA^22^.

The world population is aging and policymakers will have to face multimorbidity as a global challenge in the near future, especially in developing countries. This indicates that health care systems need to deliver early preventive and treatment programs^7^. NCDs are one of the biggest challenges for health care providers and policymakers in Eastern Mediterranean countries with a low socio-economic status, or involved in economic, demographic, geographic, or epidemiological transition. Longer-lived individuals are more likely to acquire multimorbidity, because of the increasing trend of chronic diseases in this area^4^. Therefore, studies should be conducted for the promotion of healthy lifestyles to prevent or manage multimorbidity^34^. Iran has experienced rapid urbanization, increased population age and changing disease patterns in the recent years and is now facing a new set of health care demands.

This study provides the first nationally representative estimate of NCDs multimorbidity patterns among Iranian adults. Other studies on multimorbidity conducted in other countries have included only young or middle-aged adults^35,36^, but our study included people aged from 20 to 70. In this study, the prevalence of most risk factors, such as hypertension, low physical activity, obesity, and low fruit and vegetable intake were higher in females than males.

In Ghana, India, Mexico, South Africa and the Russian Federation, the prevalence of hypertension was higher in females as well^37^. Whereas, a systematic review about blood pressure in several world countries showed that males had a higher average blood pressure than females in all world regions, expect West Africa^38^.

Higher life expectancy among females and more exposure to NCD risk factors can be one of the reasons for the higher prevalence of NCDs among females^13,39^.

In this study, the prevalence of most NCDs such as Diabetes, Coronary Artery Disease, Osteoporosis, Back Pain, Neck Pain and Knee Pain was significantly higher in females. Multimorbidity was more common among females in some previous studies^40,41^, but not is studies from Finland, Poland, and Spain^42^. A study from Kuwait also showed that the prevalence of NCDs was higher among males^43^. A potential cause for the higher prevalence of NCDs among females could be their relatively higher tendency to mention their condition in self-reports^41^ and another reason may be gender inequality in access to healthcare^44^.

In our analysis, LCA revealed four distinct subclasses of multimorbidity among the Iranian population, for males and for females. Overall, it seemed liked heterogeneity and class separation was better performed in males than females, although both genders had the same number of latent classes.

Apparently in Iran, females are more prone to develop skeletal disorders and osteoporosis, and the reason might be vitamin D deficiency and low sun exposure. Also, the reason might be that females are more susceptible, report their pain more often or are more inclined to somaticize their feelings^45,46^. In a study conducted over a 28-year period in Iran and 6 other countries, low back pain was the leading cause of YLDs (Year Lost due to Disease) in both sexes^47^. The 2016 GBD reported that the burden of disease associated with musculoskeletal health increased 19.6%, from 2006 to 2016^48^. During 1990–2013, the total DALYs of musculoskeletal disorders increased by 105.2% in the EMR, although it increased only 58.0% in the rest of the world; and low back pain and neck pain had the highest burden in EMR countries^49^. Our findings also confirm that prevention and control programmers for musculoskeletal disorders should be implemented in different levels of national health care programs.

In this study, in both genders, the chance of membership in subclasses 1 through 3 increased with age. It is evident that chronic conditions and multimorbidity typically increase with age^50^. Authors state that age is related to the duration of exposure to risk factors^37^. However, a study reported that in South Africa, multimorbidity prevalence did not increase with age and a gradual decrease after 60 years was observed^51^. The reason for this pattern might be shorter life time or mortality from communicable diseases in Africa.

In this study, in males, latent class 1 (Diabetes) was strongly associated with hypertension, physical inactivity and BMI. Hypertension increased the chance of male membership in the diabetic subclass, but in women reduced the chance of membership in the Pre-skeletal subclass. A study conducted in the population over the age of 20 years in Korea also showed an inverse relation between high blood pressure and low back pain^52^. Also a long-term cohort reported that high systolic or diastolic blood pressure was directly associated with low back pain occurrence. This association may be due to using anti-hypertensive medication and diminished pain sensitivity among patients with hypertension^53^.

In the present study, with increase in physical activity, the chance of male’s membership in the diabetic subclass decreased, but no significant association was observed in females. This may be because in Iran, males have more opportunity for doing physical activity outdoors or in their workplaces. Low physical activity is estimated to be the cause of roughly 27% of diabetes in Iran^27^. The KORA-Age Augsburg study, performed on people 65 to 94 years, also showed an inverse association between physical activity and multimorbidity among males. Multi-morbidity referred to having at least two non-communicable diseases that were self-reported. In this study, 13 non-communicable diseases were defined according to the Charlson comorbidity index^54^. Similar to these findings Alimohammadian *et al*. showed an association between decreased physical activity and multimorbidity in adults, which was more evident in males^41^. Another study from Quebec, reported no association between multimorbidity and physical activity in both genders^55^.

In the present study, increased BMI significantly increased the chance of female membership in all disease subclasses, while in males; this association was seen only in the diabetic group. Similar findings have been previously reported in several studies in which obesity was associated with multimorbidity, and obesity increased the risk diabetes and other NCDs^13,56^.

In our analysis, tobacco consumption increased the chance of both genders membership in the musculoskeletal disorders subclass, which indicates that smoking in populations might be associated with musculoskeletal pain. Studies have shown that smoking increases the risk of multimorbidity^9,13^. Studies have suggested that both smoking and female gender were independently associated with a higher odds of reporting moderate to severe pain. Also, smoking was more strongly related to increased pain among women^46^. Also a review showed that tobacco smoking has negative effects on the musculoskeletal system^57^, and cigarette smoking may be associated with decreased muscle strength. However, more research about the mechanism that smoking affects the musculoskeletal system is needed. Compared with other non-communicable diseases, impaired musculoskeletal health is responsible for the greatest loss of productive years in the workforce^48^, and requires special attention.

In this study, males with lower education were more likely to be in the diabetic and musculoskeletal disorders subclass; while in females, they were more likely to be in the asthma and peri-skeletal diseases groups. Similar findings have been previously reported from several nations, and have shown that the prevalence of multimorbidity of chronic illnesses is significantly higher among people who were older, retired and less educated^43,58^. Also in West Asia, with increased levels of education, a decrease was seen in the prevalence of multimorbidity^41^. However, in Qatar, with increasing levels of education, multimorbidity increased^59^. In this present study, at province-level, the urbanization score was not associated with non-communicable multimorbidity subclasses in females. But, urbanization increased the odds of membership in the diabetes class among males. According to GBD2017, diabetes ranked fourth as the leading cause of age-standardized YLDs in the world^47^. Adekanmbi *et al*. conducted a multilevel analysis study in Namibia and showed increased odds of diabetes among individuals with the highest urbanization and development level^60^.

In this study, similar to previous studies^41,61^, lower socioeconomic groups were associated with multimorbidity. This might partially be explained by the risk factors that the lower socioeconomic class faces such as unplanned urbanization, living in slums, physical inactivity, and consuming unhealthy food^41^. However, studies from China have shown that high income was associated with increased multimorbidity, which may be due to the high cost of health care in China^34^ which make poor families less inclined to check their health.

WHO data from low- and middle-income countries revealed a reciprocal relation between the prevalence of multimorbidity and development^62^. In high-income countries, multimorbidity has been directly associated with development^42^. Differences in the prevalence of non-communicable diseases in urban areas may be due to the impact of health services and health literacy in these areas^63^.

## Limitations

The current study had several limitations. First, this study was based on cross-sectional data which cannot show a causal relation between different risk factors and multimorbidity. Second, multimorbidity was assessed according to self-report and there is a possibility of recall bias. However, this approach is used in most national surveys, because of limited time and resources. Using self-reports also avoids potential recording errors that occur in administrative data, such as incomplete documentation, and miscoding^5^. Third, this study in comparison to the Charlson Comorbidity Index included a smaller number of diseases; and this is one reason for the few extracted classes. We suggest that future investigations include other chronic diseases such as mental disorders, kidney or liver diseases as well.

However, our study has several strengths as well. We used a large community-based sample which included adults from all provinces of Iran (3). In this study by using LCA both individual and community-level analyses was performed to describe the economic and social context in which individuals live and experience multimorbidity.

In order to control multimorbidity, health promotion and educational methods should be used to enhance public awareness about modifiable risk factors such as physical activity and smoking. These results highlight that policies, strategies and health programs for non-communicable diseases, must include musculoskeletal health as an integral component, particularly in low socioeconomic settings.

The identification of distinct subtypes of public population is important for describing the etiological pathways of morbidities which often co-occur with different NCDs. Other implications of the results of this study are helping to identify groups at risk; and can be used in developing countries with limited resources to tailor health education and services, to special groups. Also by recognizing these groups, the health system can allocate active services to them; and researchers can conduct etiologic studies by comparing these groups with other populations.

## Conclusion

The findings of this study showed that behavioral factors such as physical inactivity, tobacco use and urbanization are associated with multimorbidity in Iran; and there is a need to allocate resources for controlling, preventing and managing these risk factors. Prevention and control of multimorbidity requires health promotion programs that increase public awareness about modifiable risk factors, particularly among the at risk populations. A deeper understanding of these patterns may lead to the development of preventive measures to reduce the burden of these diseases, and offer new and comprehensive ways to manage these common conditions.
